# Circulating Levels of Persistent Organic Pollutants (POPs) and Carotid Atherosclerosis in the Elderly

**DOI:** 10.1289/ehp.1103563

**Published:** 2011-10-11

**Authors:** P. Monica Lind, Bert van Bavel, Samira Salihovic, Lars Lind

**Affiliations:** 1 Occupational and Environmental Medicine, Uppsala University, Uppsala, Sweden; 2 Man-Technology-Environment (MTM) Research Centre, Örebro University, Örebro, Sweden; 3 Department of Medicine, Uppsala University, Uppsala, Sweden

**Keywords:** atherosclerosis, atherosclerotic plaques, persistent organic pollutants (POPs), pesticides

## Abstract

Background and objective: Increased circulating levels of persistent organic pollutants (POPs) have been associated with myocardial infarction. Because myocardial infarction is an atherosclerotic disease, we investigated, in a cross-sectional study, whether POP levels are related to atherosclerosis.

Methods: In the population-based Prospective Investigation of the Vasculature in Uppsala Seniors (PIVUS) study (*n* = 1,016 participants 70 years of age), the prevalence of carotid artery plaques was determined by ultrasound. The number of carotid arteries with plaques (0, 1, or 2) was recorded. Also, the intima-media thickness (IMT) and gray scale median of the intima-media complex (IM-GSM) were measured. Twenty-three POPs, comprising 16 polychlorinated biphenyls (PCBs), 5 pesticides, 1 dioxin, and 1 brominated compound (brominated diphenyl ether congener BDE-47), were analyzed by high-resolution chromatography coupled to high-resolution mass spectrometry.

Results: Seven of the POPs (PCB congeners 153, 156, 157, 170, 180, 206, and 209) were significantly associated with the number of carotid arteries with plaques even after adjusting for multiple risk factors (sex, waist circumference, body mass index, fasting blood glucose, systolic and diastolic blood pressure, high-density lipoprotein and low-density lipoprotein cholesterol, serum triglycerides, smoking, antihypertensive treatment, and statin use; *p* = 0.002–0.0001). Highly chlorinated PCBs (congeners 194, 206, and 209) were associated with an echolucent IM-GSM (*p* < 0.0001 after adjustment), whereas associations between POPs and IMT were modest.

Conclusions: Circulating levels of PCBs were associated with atherosclerotic plaques and echogenicity of the intima-media complex independent of cardiovascular risk factors, including lipids. This suggests that POPs may be a risk factor for myocardial infarction, but associations need to be confirmed in prospective studies.

Persistent organic pollutants (POPs) are chemical substances that persist in the environment, bioaccumulate through the food web, and pose a risk of causing adverse effects to human health and the environment. There is evidence of long-range transport of these substances to regions where they have never been used or produced, resulting in exposure of most human populations to POPs through consumption of fat-containing food such as fish, dairy products, and meat (Letcher et al. 2010).

During recent years, elevated circulating levels of POPs have been associated with a number of cardiovascular (CV) risk factors, such as hypertension, obesity, and diabetes, as well as with metabolic syndrome (Everett et al. 2008; Ha et al. 2009; Lee et al. 2007, 2011b; Rönn et al. 2011; Uemura et al. 2009). Furthermore, circulating levels of POPs have been associated with a history of myocardial infarction (Flesch-Janys et al. 1995; Sergeev and Carpenter 2005).

A characteristic feature of myocardial infarction is atherosclerosis. Because coronary and carotid artery atherosclerosis often go hand in hand (Hulthe et al. 1997), we hypothesized that participants with high levels of circulating POPs would have a higher incidence of carotid atherosclerotic plaques. Furthermore, because we recently found that the echogenicity of the intima-media complex in the carotid artery, a possible marker of lipid infiltration in the vascular wall, is a predictor of future CV death (Wohlin et al. 2009), we also investigated whether POP levels are associated with the gray scale median of the carotid artery intima-media complex (IM-GSM). To test these hypotheses, we used the population-based Prospective Investigation of the Vasculature in Uppsala Seniors (PIVUS) study (Lind et al. 2005), from which we have data on atherosclerosis and circulating POP levels for almost 1,000 participants.

Specifically, we tested associations between POP levels and the prevalence of overt carotid plaques, and intima-media thickness (IMT; an early marker of atherosclerosis development measurable in participants without overt plaque). In addition, we tested associations with carotid artery IM-GSM, a marker of lipid infiltration in the vascular wall that might be altered even before a plaque could be measured.

## Materials and Methods

*Participants.* Eligible participants were all 70 years of age and lived in the community of Uppsala, Sweden. The participants were randomly chosen from the register of persons living in the community. A total of 1,016 persons participated, giving a participation rate of 50.1%. The study was approved by the Ethics Committee of the University of Uppsala, and all the participants gave their informed consent before the study.

All participants were evaluated in the morning after an overnight fast. No medication or smoking was allowed after midnight. The participants were asked to answer a questionnaire about their medical history, smoking habits, and current use of regular medication.

We measured the blood pressure in the noncannulated arm of each participant using a calibrated mercury sphygmomanometer. Blood pressure was measured to the nearest 1 mmHg after at least 30 min of rest, and the average of three recordings was used. Lipid variables and fasting blood glucose were measured by standard laboratory techniques.

**Figure 1:**
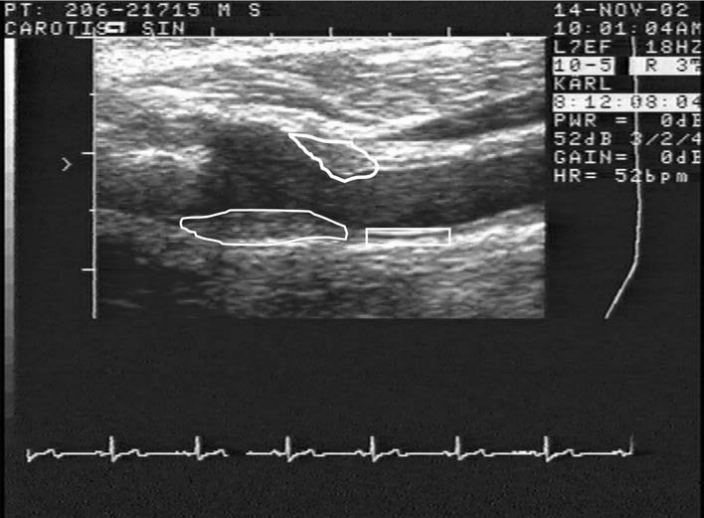
An ultrasonographic image of the left carotid artery from a study participant. The blood inside the artery appears black. The right side of the image shows the part of the common carotid artery where IMT and IM-GSM are measured (rectangle). IMT is the thickness of that part of the vascular wall, and IM-GSM is a measure of the echogenicity (gray scale) of the same part of the wall. At the left side of the image, the carotid artery has divided into an internal part and an external part. In the bifurcation region, two plaques are outlined.

*Ultrasound evaluation of the carotid artery.* The carotid artery was assessed by external B-mode ultrasound imaging (Acuson XP128 with a 10 MHz linear transducer; Acuson Corp, Mountain View, CA, USA). We visually inspected the common carotid artery, the bulb, and the internal carotid artery at both sides for the presence of plaque. A plaque was judged to be present in a particular carotid artery if a local thickening of the IMT was seen that was > 50% thicker than the surrounding IMT in any part of the carotid artery investigated. We recorded the presence of carotid plaques in none, one, or both of the carotid arteries (Figure 1).

The images were digitized and imported into the automated Artery Measurement Software, version 1.0 (Liang et al. 2000) for dedicated analysis of IMT and the IM-GSM. For IMT analysis, a maximal 10-mm segment with good image quality was chosen from the common carotid artery close to the bifurcation point. A region of interest was placed manually around the intima-media complex, which was evaluated for IMT. The program calculated the echogenicity in the intima-media complex by analyzing individual pixels within the region of interest on a scale from 0 (black) to 256 (white). Thus, a low IM-GSM value indicated a lipid-rich wall, while a high value represented a vascular wall rich in collagen or calcium. Like IMT, the gray scale median value given is the mean value from right and left carotid arteries. We repeated the IMT measurements in 30 random participants, giving a coefficient of variation for carotid artery IMT of 7.2% and for IM-GSM of 7.5%.

*Method for analyses of POPs.* POPs were measured in stored serum samples collected at baseline. We used a sample cleanup and extraction method based on a method by Sandau et al. (2003), with some modifications. Briefly, 1 mL formic acid was added to 0.5 mL plasma sample and sonicated. Labeled ^13^C internal standards and 1 mL 3% isopropanol in water were added after 60 min, followed by another sonication. Solid-phase extraction (SPE) was performed by loading the sample on a conditioned Oasis® HLB SPE (Waters, Milford, MA, USA) cartridge (6 cm^3^/150 mg). The cartridge was rinsed with 6 mL 3% isopropanol in water and 6 mL 40% methanol in water. After drying under nitrogen, the target compounds were eluted with 6 mL methylene chloride:hexane (1:1). Further cleanup was performed using a small, activated multilayer silica gel column (2 mL, 1.5 g) eluted with 7.5 mL hexane. After evaporation and addition of the ^13^C-labeled recovery standard, the volume was reduced to 25 μL tetradecane. The final measurements were performed on a Micromass Autospec Ultima (Waters) high-resolution gas chromatograph/high-resolution mass spectrometer. We monitored the two most abundant ions of the chlorine or bromine cluster in addition to one ion for the ^13^C-labeled internal and recovery standards by injecting 2 μL on a 6890N gas chromatograph (Agilent Technologies, Atlanta, GA, USA) containing a 30 m × 0.25 i.d. × 0.25 μm DB-5 capillary column (SGE Analytical Science, Victoria, AUS).

*Quality assurance/quality control.* Quality control plasma samples and procedural blank samples were incorporated in each batch of 10 samples. Blank samples did not contain any target compounds at levels > 5% of the levels in the samples except for *cis*-chlordane and *trans*-chlordane. The recoveries of the internal standards were satisfactory, in general ranging from 60–110%. The relative standard deviation of the 100 quality assurance/quality control (QA/QC) samples was < 25% for all compounds except for the compounds that were present at low levels and just above the limit of detection (LOD) in the QA/QC sample. The laboratory routinely takes part in international laboratory comparison studies, with good results (*z*-scores < 2).

Tables 1 and 2 provide a detailed description of the POPs with detection rates, LODs, and distribution.

**Table 1:** Selected basic characteristics and major CV risk factors in the PIVUS study (*n* = 1,016; 50.2% females).

Variable	Mean ± SD or *n* (%)	Median (range)
Height (cm)		169 ± 9.1		169 (151–190)
Weight (kg)		77 ± 14		76 (49–116)
Waist circumference (cm)		91 ± 12		90 (67–123)
BMI (kg/m^2^)		27.0 ± 4.3		26.6 (19.1–39)
Waist:hip ratio		0.90 ± 0.075		0.90 (0.73–1.08)
SBP (mmHg)		150 ± 23		148 (105–210)
DBP (mmHg)		79 ± 10		78 (56–105)
Serum cholesterol (mmol/L)		5.4 ± 1.0		5.4 (3.2–7.8)
LDL cholesterol (mmol/L)		3.3 ± 0.88		3.3 (1.4–5.6)
HDL cholesterol (mmol/L)		1.5 ± 0.42		1.4 (0.8–2.9)
Serum triglycerides (mmol/L)		1.3 ± 0.60		1.15 (0.46–3.6)
Fasting blood glucose (mmol/L)		5.3 ± 1.6		5 (3.8–13.5)
IMT		0.88 ± 0.16		0.87 (0.58–1.35)
IM-GSM		78.8 ± 23.8		75.5 (37–143.5)
Current smoking		107 (11)		—
History of myocardial infarction		72 (7)		—
History of stroke		37 (4)		—
History of diabetes mellitus		88 (9)		—
Any CV medication		461 (45)		—
Antihypertensive medication		317 (31)		—
Statin treatment		149 (15)		—
Insulin therapy		18 (2)		—
Oral antiglycemic drug therapy		62 (6)		—
No carotid artery plaque		327 (35)		—
Unilateral carotid plaque		343 (36)		—
Bilateral carotid plaque		273 (29)		—

**Table 2:** Serum concentrations (pg/mL) of POPs in the PIVUS study (*n* = 1,016; 50.2% females).

POP	Detection rate (%)	LOD	POP concentration [median (25th–75th percentile)]
PCBs						
PCB-74 (2,2´,4,4´,5-pentachlorobiphenyl)		100		8.5		91.4 (63.9–128.1)
PCB-99 (2,2´,4,4´,5-pentachlorobiphenyl)		99.5		10.0		90.9 (62.4–131.9)
PCB-105 (2,3,3´,4,4´-pentachlorobiphenyl)*a*		100		5.9		32.0 (21.0–46.8)
PCB-118 (2,3´,4,4´,5-pentachlorobiphenyl)*a*		100		25.3		200.6 (136.4–281.0)
PCB-126 (3,3´,4,4´,5-pentachlorobiphenyl)*b*		95.6		8.0		40.4 (71.9–385.8)
PCB-138 (2,2´,3,4,4´,5´-hexachlorobiphenyl)		99.6		108.7		819.3 (619.2–1115.8)
PCB-153 (2,2´,4,4´,5,5´-hexachlorobiphenyl)		100		117.7		1427.6 (1114.4–1847.9)
PCB-156 (2,3,3´,4,4´,5-hexachlorobiphenyl)*a*		100		10.8		154.3 (118.7–197.6)
PCB-157 (2,3,3´,4,4´,5´-hexachlorobiphenyl)*a*		100		1.5		28.0 (21.4–37.0)
PCB-169 (3,3´,4,4´,5,5´-hexachlorobiphenyl)*b*		99.7		17.5		171.4 (130.1.9–636.4)
PCB-170 (2,2´,3,4,4´,5,5´-heptachlorobiphenyl)		100		38.9		497.5 (385.7–633)
PCB-180 (3,3´,4,4´,5,5´-hexachlorobiphenyl)		100		65.3		1165.4 (917.8–1487.8)
PCB-189 (2,3,3´,4,4´,5,5´-heptachlorobiphenyl)*a*		100		1.7		19.3 (14.6–25.8)
PCB-194 (2,2´,3,3´,4,4´,5,5´-octachlorobiphenyl)		98.6		4.2		119.4 (87.6–158.9)
PCB-206 (2,2´,3,3´,4,4´,5,5´,6-nonachlorobiphenyl)		100		0.8		26.8 (20.8–35.2)
PCB-209 (2,2´,3,3´,4,4´,5,5´,6,6´-decachlorobiphenyl)		100		1.2		26.2 (19.6–34.7)
OCDD		80.6		1.4		2.6 (1.4–4.2)
OC pesticides						
HCB		98.6		89.1		253.6 (189.1–336.5)
*cis*-Chlordane		9.6		—		< LOD
*trans*-Chlordane		3.4		—		< LOD
*trans*-Nonachlordane (TNC)		100		5.9		139.6 (91.6–211.4)
DDE		100		11.5		1859.8 (1024.0–3472.1)
BDE-47 (2,2´,4,4´-tetra-bromodiphenyl ether)		77.2		9.2		12.6 (9.0–19.5)
**a**Dioxin-like PCB (mono-*ortho* substituted). **b**Dioxin-like PCB (coplanar, non-*ortho* substituted).						

A total toxic equivalency (TEQ) value was calculated for the polychlorinated biphenyls (PCBs) as well as octachlorodibenzo-*p*-dioxin (OCDD) according to Van den Berg et al. (1998). TEQ was also calculated separately for the dioxin-like non-*ortho*-PCBs (congeners 126 and 169) and the dioxin-like mono-*ortho*-PCBs.

*Statistical analysis.* POPs were measured in 992 participants; data were missing for some participants because of a lack of plasma samples. IMT and IM-GSM were evaluated in 990 participants, while plaque prevalence was evaluated in only 943 participants because of bad image quality for some participants. Data for individual model covariates used to adjust for potential confounding were missing for < 10 participants.

We evaluated all variables for nonnormality. Variables with a skewed distribution, including fasting glucose, serum triglycerides, all POPs, the sum of the PCBs, and TEQ, were natural log (ln) transformed. In addition, the POPs were divided into quintiles to evaluate potential nonlinear relationships.

Ordinal logistic regression models were used when the outcome was number of carotid arteries with plaques (grouped as 0, 1, or 2; the dependent variable). Linear regression was used when the outcomes were the continuous variables IMT or IM-GSM (the dependent variable). Linear and logistic regression modeling was done according to Dobson (2002).

We evaluated all interactions between the POP levels and sex for all POPs and for all outcomes by introducing an interaction term between the POP under investigation and sex together with the POP and sex terms. Because no such interactions (*p* < 0.05) were found, sex adjustment was performed in the first set of models.

In the second sets of models, we adjusted for multiple CV risk factors [sex, waist circumference, body mass index (BMI), ln-transformed fasting blood glucose, systolic blood pressure (SBP), diastolic blood pressure (DBP), high-density lipoprotein (HDL) and low-density lipoprotein (LDL) cholesterol, ln-transformed serum triglycerides, current smoking, antihypertensive treatment, and statin use].

In all the above models, analyses were first run with the POPs modeled as ln-transformed continuous variables and categorized using quintiles. Because of the large number of POPs analyzed, we applied a Bonferroni correction to determine the alpha level for statistical significance (0.05/21 = 0.00238) for each of the three outcomes: plaque occurrence, IMT, and IM-GSM. The software used was STATA (version 11; StataCorp, College Station, TX, USA).

## Results

Approximately 7% of the cohort reported a history of myocardial infarction, 4% stroke, and 9% diabetes mellitus. Almost half the cohort (45%) reported use of CV medication, with antihypertensive medication being the most common (32%). Fifteen percent of the participants reported use of statins. Two percent of the sample were on insulin therapy, and 6% were on regular oral antiglycemic drugs (Table 1; for details, see Lind et al. 2005).

A total of 23 POPs were measured: 16 PCB congeners, 5 organochlorine (OC) pesticides, 1 OCDD, and 1 brominated diphenyl ether (BDE) congener. We evaluated POPs that we found at detectable levels in > 90% of the study population. Because the statistical analysis is limited by a large number of cases with undetectable levels, two OC pesticides (*trans*-chlordane and *cis*-chlordane) with detection rates < 10% were excluded from the final analyses (Table 2).

*POPs versus carotid plaques.* Models of ln-transformed POP levels as continuous variables identified 7 POPs (PCB congeners 153, 156, 157, 170, 180, 206, and 209) that were significantly associated (*p* < 0.00238) with the number of carotid arteries with plaques, even after adjusting for sex or sex and multiple CV risk factors (Table 3).

**Table 3:** Relationships among POPs, plaques in the carotid artery, and echogenicity IM-GSM in the common carotid artery (ln-transformed continuous variables).

Carotid plaques			IM-GSM			IMT		
POP	OR (95% CI)	Multiple adjusted *p*-value*a*	Regression coefficient (95% CI)	Multiple adjusted *p*-value*a*	Regression coefficient (95% CI)	Multiple adjusted *p*-value*a*
PCB-74		1.28 (1.00, 1.63)		0.054		2.3 (–0.71, 5.29)		0.14		0.15 (–0.005, 0.035)		0.17
PCB-99		1.29 (1.04, 1.61)		0.02		0.41 (–2.19, 3.01)		0.76		0.001 (–0.016, 0.019)		0.87
PCB-105*b*		1.07 (0.86, 1.33)		0.53		0.77 (–1.88, 3.43)		0.57		0.0016 (–0.016, 0.019)		0.86
PCB-118*b*		1.16 (0.88, 1.44)		0.31		0.69 (–2.25, 3.63)		0.65		0.002 (–0.018, 0.022)		0.84
PCB-126*c*		1.06 (0.93, 1.21)		0.44		–3.26 (–4.92, –1.61)		0.0001		0.013 (0.002, 0.024)		0.022
PCB-138		1.46 (1.11, 1.93)		0.007		–1.24 (–4.54, 2.05)		0.46		0.008 (–0.014, 0.030)		0.46
PCB-153		1.65 (1.22, 2.25)		0.001		–0.10 (–3.72, 3.53)		0.96		0.011 (0.013, 0.035)		0.38
PCB-156*b*		1.95 (1.41, 2.71)		0.0001		1.41 (–2.41, 5.23)		0.47		0.001 (–0.025, 0.026)		0.95
PCB-157*b*		1.56 (1.17, 2.06)		0.002		–1.41 (–4.80, 1.97)		0.42		0.034 (0.011, 0.057)		0.003
PCB-169*c*		1.18 (0.87, 1.59)		0.28		–1.66 (–5.07, 1.75)		0.34		0.002 (–0.022, 0.026)		0.86
PCB-170		2.02 (1.43, 2.85)		0.0001		–1.93 (–6.04, 2.18)		0.36		0.022 (–0.006, 0.050)		0.13
PCB-180		1.86 (1.33, 2.61)		0.0001		–3.17 (–7.21, 0.87)		0.12		0.024 (–0.004, 0.051)		0.09
PCB-189*b*		1.15 (0.95, 1.39)		0.15		–0.75 (–3.11, 1.60)		0.53		0.007 (–0.009, 0.023)		0.41
PCB-194		1.14 (0.97, 1.33)		0.102		–10.30 (–12.13, –8.47)		0.0001		0.016 (0.002, 0.029)		0.020
PCB-206		1.66 (1.24, 2.23)		0.001		–9.64 (–13.09, –6.18)		0.0001		0.031 (0.007, 0.055)		0.012
PCB-209		1.51 (1.16, 1.95)		0.002		–13.24 (–16.30, –10.77)		0.0001		0.026 (0.004, 0.048)		0.018
OCDD		1.31 (1.06, 1.62)		0.01		2.97 (0.39, 5.55)		0.024		–0.017 (–0.019, 0.016)		0.85
HCB		1.20 (0.90, 1.60)		0.21		9.01 (5.49, 12.52)		0.0001		–0.001 (–0.024, 0.024)		0.99
TNC		1.33 (1.07, 1.66)		0.010		6.29 (3.67, 8.91)		0.0001		–0.002 (–0.020, 0.016)		0.82
DDE		1.02 (0.89, 1.17)		0.80		–2.53 (–4.21, –0.86)		0.003		–0.004 (–0.015, 0.007)		0.49
BDE-47		1.12 (0.93, 1.34)		0.24		2.42 (0.17, 4.67)		0.035		–0.008 (–0023, 0.0073)		0.31
Sum of PCBs (concentration)		1.03 (1.01, 1.05)		0.002		–0.40 (–0.65, –0.14)		0.002		0.001 (–0.00004, 0.0033)		0.057
Total TEQ		1.09 (0.90, 1.33)		0.34		–4.83 (–7.15, –2.52)		0.0001		0.021 (0.0052, 0.037)		0.009
TEQ for dioxin-like mono-*ortho*-substituted PCBs*b*		1.67 (1.20, 2.32)		0.002		1.06 (–2.88, 5.02)		0.60		0.010 (–0.016, 0.037)		0.44
TEQ for dioxin-like coplanar, non‑*ortho*-substituted PCBs*c*		1.09 (0.90, 1.32)		0.36		–4.50 (–6.79, –2.21)		0.0001		0.020 (0.0045, 0.037)		0.012
Penta-PCBs (concentration)		1.04 (0.98, 1.11)		0.12		–0.52 (–1.26, 0.21)		0.16		0.0029 (–0.0020, 0.0079)		0.25
Hexa-PCBs (concentration)		1.12 (1.05, 1.21)		0.001		–0.22 (–1.04, 0.60)		0.60		0.0034 (–0.0021, 0.0089)		0.23
Hepta-PCBs (concentration)		1.17 (1.06, 1.30)		0.0001		–0.68 (–1.91, 0.55)		0.28		0.0059 (–0.0024, 0.014)		0.16
Octa- to deca-PCBs (concentrations)		1.11 (1.03, 1.20)		0.007		–4.71 (–5.65, –3.77)		0.0001		0.0090 (0.0022, 0.015)		0.009
**a***p*-Values are adjusted for sex, waist circumference, BMI, fasting blood glucose, SBP, DBP, HDL and LDL cholesterol, serum triglycerides, smoking, antihypertensive treatment, and statin use. **b**Dioxin-like PCB (mono-*ortho* substituted). **c**Dioxin-like PCB (coplanar, non-*ortho* substituted).												

No significant interactions were seen between POP levels and sex regarding carotid plaque except for hexachlorobenzene (HCB). For HCB, however, no statistically significant relationships with carotid atherosclerosis were seen after adjusting for multiple testing [after adjusting for multiple risk factors; men: odds ratio (OR) = 0.92; 95% confidence interval (CI): 0.60, 1.41; women: OR = 1.5; 95% CI: 1.01, 2.24]. For the other POPs, we adjusted, rather than stratified, the sample for sex.

When we divided the POP levels into quintiles, we found monotonic relationships versus the number of arteries with plaques for the POPs described above (data not shown).

A significant relationship was found between the ln-transformed sum of the PCBs and the number of arteries with plaques after adjusting for multiple risk factors (OR = 1.03; 95% CI: 1.01, 1.05; *p* = 0.002). When we summed subsets of PCBs according to the number of chlorine atoms, PCBs with fewer than six chlorine atoms were not related to carotid plaques, whereas those with six or more chlorine atoms were significantly associated with plaque prevalence (Table 3).

Total TEQ (ln-transformed) was not significantly associated with the number of arteries with plaques. Nonetheless, TEQ for the dioxin-like mono-*ortho*-PCBs, but not for the dioxin-like non-*ortho*-PCBs, was significantly related to plaque prevalence (Table 3).

*POPs versus IMT.* No single POP was related to IMT after adjusting for multiple risk factors and multiple testing (Table 3). No evidence was seen of any low-dose effect, as defined by a marked change in the median for IMT already taking place at the second quartile (data not shown).

The sum of the PCBs was not significantly associated with IMT after adjusting for multiple risk factors. The sum of PCBs with fewer than 8 chlorine atoms also was not associated with IMT, but the sum of PCBs with 8–10 chlorine atoms was significantly related to IMT (*p* = 0.009; Table 3). Total TEQ was associated with IMT after adjusting for multiple risk factors (*p* = 0.009; Table 3). The TEQ for the dioxin-like non-*ortho*-PCBs, but not for the dioxin-like mono-*ortho*-PCBs, was also significantly associated with IMT.

*POPs versus IM-GSM.* IM-GSM was inversely associated with ln-transformed PCB-126 and the highly chlorinated PCBs (congeners 194, 206, and 209) after adjusting for multiple risk factors (*p* < 0.0001; Table 3) and positively associated with HCB and *trans*-nonachlordane (TNC; *p* < 0.0001). The inverse relationship between 1,1-bis-(4-chlorophenyl)-2,2-dichloroethene (DDE) levels and IM-GSM approached significance after adjusting for multiple risk factors and multiple testing (*p* = 0.003).

No significant interactions were seen between the POP levels and sex regarding IM-GSM. Consequently, we adjusted, rather than stratified, the sample for sex. When the POP levels were divided into quintiles, monotonic relationships were seen between IM-GSM and PCBs congeners 206 and 209 (data not shown).

An inverse significant relationship was found between the sum of the PCBs and IM-GSM after adjusting for multiple risk factors (*p* = 0.002; Table 3). When the sum of PCBs was divided according to the number of chlorine atoms, PCBs with fewer than 8 chlorine atoms were not associated with IM-GSM; however, PCBs with 8–10 chlorine atoms had a significant inverse association with IM-GSM (*p* = 0.0001). Total TEQ was inversely related to IM-GSM after adjusting for multiple risk factors (*p* = 0.0001). However, only TEQ for the dioxin-like non-ortho-PCBs, not for the dioxin-like *mono*-ortho-PCBs, was significantly related to IM-GSM.

*Stratification by CV medication.* To investigate whether the observed associations reported above were similar in participants with CV medication and those without, we performed the analysis again in participants with CV medication (45% of the sample) and participants without CV medication separately. In general, estimated associations according to medication use were comparable for all three outcomes with those estimated for the population as a whole. One exception was the association between OCDD and IM-GSM, which was evident only in participants not using CV medication (OR = 1.51; 95% CI: 1.41, 2.01; vs. participants using CV medication: OR = 1.00; 95% CI: 0.74, 1.37).

## Discussion

In the present cross-sectional study, several of the individual PCBs, as well as the sum of the PCBs, were associated with the presence of carotid artery plaques. Some of the highly chlorinated PCBs were also associated with the echogenicity of the intima-media complex, a marker of vascular wall composition. Total TEQ was positively associated with IMT and inversely associated with the echogenicity of the intima-media complex but not associated with plaque prevalence. These relationships were statistically significant after adjusting for CV risk factors, including lipids, suggesting that POPs may have a vascular effect not mediated by traditional risk factors for atherosclerosis. To the best of our knowledge, no other study has reported associations between POP levels and measures of atherosclerosis in a large human sample.

*Comparison with the literature.* Associations between POP exposure and myocardial infarction based on studies of highly exposed individuals (through accident or occupational exposure) and studies of representative population-based samples have been reported (Chase et al. 1982; Dalton et al. 2001; Sergeev and Carpenter 2010). However, because POP exposure has also been associated with well-known CV risk factors such as hypertension, diabetes, hyperlipidemia, and obesity (Everett et al. 2008; Goncharov et al. 2008; Lee et al. 2007, 2011a, 2011b; Rönn et al. 2011), it is unclear whether POPs might induce myocardial infarction by altering risk factors or by a more direct action on the vascular wall.

*Biological rationale.* Development of atherosclerosis is a complicated chain of events starting with accumulation of LDL cholesterol in the subintimal space. After oxidation of LDL, immunocompetent cells such as monocytes and T-cell lymphocytes are attracted to the site of lipid oxidation by activation of adhesion molecules from the endothelium, and the chronic repair-of-injury process begins with the liberation of proinflammatory cytokines.

POPs may interfere with this process in several ways. For example, POPs have been associated with an increased blood pressure, with proatherogenic alterations in lipid metabolism, and with the development of diabetes and obesity (Everett et al. 2008; Ha et al. 2009; Lee et al. 2007, 2011b; Rönn et al. 2011; Uemura et al. 2009). However, associations between several of the POPs and markers of atherosclerosis were evident despite adjustment for classical CV risk factors, which suggests that associations, if causal, might reflect direct effects of POPs on atherosclerosis. Moreover, POPs have been found to impair endothelium-dependent vasodilatation in mice (Kopf et al. 2008) and alter the expression of genes involved in DNA repair and cell cycling in vascular smooth muscle cells (Puga et al. 2004). Several POPs activate the cytosolic aryl hydrocarbon receptor (AHR). For example, activation of the AHR by dioxin induces an oxidative stress response (Kopf et al. 2008; Park et al. 1996; Shertzer et al. 1998), which might contribute to atherosclerosis via the oxidation of LDL, an early event in the formation of atherosclerotic plaques. Activation of the AHR also triggers up-regulation of genes such as cytochrome P450 1A1 (*CYP1A1*) and cyclooxygenase-2 (*COX2*), whose products metabolize arachidonic acid into vasoactive eicosanoids known to be involved in the atherosclerotic process (Dalton et al. 2001; Kraemer et al. 1996; Puga et al. 1997, 2004). Cross talk between the AHR and sex-hormone receptors is a well-known occurrence (Ohtake et al. 2008), and it is also well known that estrogen and testosterone are important players in atherosclerosis (Bourghardt et al. 2010; Kolovou et al. 2011). We have recently reported an association between circulating levels of the estrogen-receptor ligand bisphenol A and atherosclerosis in the present study population (Lind and Lind 2011).

Other mechanisms may exist whereby POPs could influence several of the major stages in the atherosclerotic process. The association between TEQ and atherosclerosis in the present study, together with a previous experimental finding that tetrachlorodibenzo-*p*-dioxin increases the formation of plaques in apolipoprotein E knockout mice, a well-known model of atherosclerosis formation (Dalton et al. 2001), supports the involvement of the AHR in atherosclerosis formation.

*Different measurements of atherosclerosis.* In the present study, we evaluated three markers that reflect different characteristics of the atherosclerotic process: plaque prevalence, IMT, and IM-GSM. All three measures are related to future CV events (Davidsson et al. 2010; O’Leary et al. 1999; Wohlin et al. 2009), but IMT and IM-GSM are measured in the plaque-free part of the vascular wall and consequently represent processes that occur before overt plaques form. However, IMT and IM-GSM are poorly correlated with each other (*R*^2^ < 0.01), and although the major risk factors for IMT in the present cohort were SBP and smoking, the major risk factors for IM-GSM were lipids and markers of inflammation and oxidative stress (Andersson et al. 2009). This suggests that the two measures, IMT and IM-GSM, may reflect different aspects of atherosclerosis development that may be affected in different ways by POPs. Furthermore, although IMT and overt plaque formation were both associated with blood pressure and smoking in the study population, markers of oxidative stress and inflammation were more closely associated with plaque than with IMT (Andersson et al. 2009). This may explain why POPs could be more closely related with plaque prevalence than with IMT, i.e., because activation of the AHR could induce oxidative stress and inflammation (Park et al. 1996; Shertzer et al. 1998).

*Carotid artery plaque.* The presence of atherosclerotic plaques in the carotid arteries is a known predictor of future CV events (O’Leary et al. 1999). Atherosclerosis in the carotid arteries is also predictive of plaques in the coronary circulation (Hulthe et al. 1997). Consequently, persons with carotid artery plaques are at increased risk for myocardial infarction. Furthermore, because persons with bilateral carotid plaques are at higher risk of future CV disorders compared with those with unilateral plaques (Davidsson et al. 2010), we used a graded response for carotid plaques (0, 1, or 2 carotid arteries with plaques) rather than a binary outcome (plaque or no plaque) for our study participants.

*Echogenicity of the intima-media complex.* The echogenicity of the intima-media complex is closely related to the echogenicity of overt plaques (Lind et al. 2007) and has been shown to be a powerful predictor of future CV mortality (Wohlin et al. 2009). The echogenicity of overt plaques is related to the composition of the plaque (El-Barghouty et al. 1996), and it is therefore likely that the echogenicity of the intima-media complex is also a marker for the composition of the vascular wall.

In the present study, the highly chlorinated PCBs were associated with an echolucent intima-media complex, a likely marker for a lipid-rich vascular wall, even after adjusting for CV risk factors, including lipids. By contrast, HCB and TNC were associated with an echogenic intima-media complex, suggesting that these pesticides might affect vascular walls with compositions opposite to those affected by highly chlorinated POPs. In contrast to the findings for plaques, total TEQ was associated with IM-GSM, supporting an AHR-mediated effect of the PCBs on this outcome.

*Intima-media thickness.* IMT is a known predictor of CV events and is usually regarded as a marker of atherosclerosis (O’Leary et al. 1999). Atherosclerosis is a disorder of the intima, but IMT also incorporates the media in the measurement and thus might distort the relationship with POP levels.

*Nonlinear relationships.* In most cases, models of POPs categorized by quintiles supported linear relationships with atherosclerosis. It has been argued that nonmonotonic, low-dose effects could be a characteristic feature of many POPs (Gregoraszczuk et al. 2008; Haase et al. 2009; Kim et al. 2010; Lee et al. 2007), but the present study produced very little evidence to support this.

*Adjustment for lipids.* Traditionally, POP levels are normalized for lipids and given per gram lipid because these compounds are lipid soluble (Bernert et al. 2007). However, this normalization may not be appropriate when dealing with CV diseases because POP exposure has been shown to alter lipid levels in both human and animal studies (Bell et al. 1994; Goncharov et al. 2008; Kanagawa et al. 2008; Kratz 2005; Lind et al. 2004). We therefore chose to use wet-weight levels for the POPs but adjusted for lipids (together with the multiple risk factors) in the second model. It has also been argued that lipid normalization tends to overadjust for the effects of lipids and that adjusting the statistical models is more appropriate (Gaskins and Schisterman 2009). This lipid adjustment, however, only marginally changed the results in the present study. Furthermore, essentially the same results as presented above were obtained when we used lipid-normalized POP levels in the analysis (data not shown).

*Limitations of the study.* The cross-sectional study population was limited to Caucasians 70 years of age, which means that caution should be taken about generalizing to other ethnic and age groups.

The present study had a moderate participation rate. However, an analysis of nonparticipants showed the present sample to be fairly representative of the total population regarding most CV disorders and drug intake (Lind et al. 2005).

## Conclusions

Circulating levels of PCBs were associated with atherosclerotic plaques and the echogenicity of the intima-media complex independent of CV risk factors, including lipids. This suggests an effect of POPs on the risk of myocardial infarction that should be investigated in prospective studies.
